# The COVID-19 pandemic and its possible impact on the treatment of odontogenic and intraoral abscesses

**DOI:** 10.1186/s13005-023-00381-2

**Published:** 2023-08-19

**Authors:** Florian D. Grill, Paulina Rothlauf, Lucas M. Ritschl, Herbert Deppe, Herbert Stimmer, Florian Scheufele, Matthias Schwarz, Klaus-Dietrich Wolff, Andreas M. Fichter

**Affiliations:** 1 Department of Oral and Maxillofacial Surgery, School of Medicine, Technical University of Munich, Ismaninger Str. 22, Munich, 81675 Germany; 2 Department of Diagnostic and Interventional Radiology, School of Medicine, Technical University of Munich, Munich, Germany; 3 Department of Surgery, School of Medicine, Technical University of Munich, Munich, Germany

**Keywords:** Odontogenic abscess, Covid-19, Lockdown

## Abstract

Most odontogenic and intraoral abscesses can be treated on an outpatient basis with local anesthesia. However, severe disease progression may require an incision under general anesthesia (GA) with postoperative inpatient treatment. This study aimed to evaluate the first “COVID-19 year” in Germany and compare the first “COVID-19 year” with the two previous years. All consecutive cases with odontogenic or intraoral abscesses treated in an outpatient or inpatient setting between 2018 and 2021 were included in this study. Data were collected, including the type of anesthesia, length of hospital stay, and healthcare costs. Despite the lower total number of abscess treatments in the first year of COVID-19 (*n* = 298 patients) than that in the two previous years (*n* = 663 patients), the number of advanced abscesses requiring intervention under GA was significantly higher (*p* < 0.001). This increased burden of care was also reflected in increased healthcare costs. The measures taken against the COVID-19 pandemic had an impact on the course of other diseases, for example, odontogenic and intraoral abscesses. The results showed an emerging conflict in patient care during the pandemic crisis that should be considered in possible future pandemics.

## Introduction

Odontogenic infections are commonly caused by dental caries and usually progresses through three phases: inoculation, cellulitis, and abscess formation [1, 18, 28]. Furthermore, the prevalence of odontogenic infections is quite high, with a reported incidence of 5–7 per 100,000 requiring hospital care in Finland [23]. The causes of abscesses are well-known and include caries, trauma, and periodontal lesions [3, 11]. If left untreated in the early stages, the abscess can spread and develop into a compartmental abscess. This can lead to severe complications and is potentially life-threatening. Abscess treatment involves incision and drainage. Treatment under local anaesthesia can be performed in localised abscesses such as paramandibular abscesses without further clinical compromise such as breathing or swallowing problems [1]. If the abscess formation cannot be reached intraorally due to involvement of fascial spaces and/or in cases of severe trismus, airway compression and multiple sites, an extraoral incision must be performed [9]. Advanced abscess formations with a tendency to expand, especially in combination with systemic complications and potentially life-threatening disease courses, are indicated for inpatient treatment with intravenous antibiotic administration [1].

In late 2019 and early 2020, the world’s attention was drawn to the emergence of the COVID-19 virus. SARS-CoV-2 was identified as the causative agent for the severe acute respiratory syndrome coronavirus-2, which gave rise to coronavirus disease 2019 (COVID-19) and developed into a global threat. The virus itself is a novel enveloped beta-coronavirus with a single-stranded positive-sense RNA, larger than other RNA viruses with a genome size of 8.4–12 kDa. The virus is commonly transmitted via respiratory droplets [12, 27, 30].

On January 30, 2020, the World Health Organization characterized the situation a Public Health Emergency of International Concern, and on March 11, 2020, it declared COVID-19 a pandemic [31]. As a result of the increasing number of registered infections, the German federal administration decided to impose a lockdown with extensive restrictions on personal contacts and public life [4]. In the southern German state of Bavaria, where our department is located, the lockdown restrictions were eased on May 4, 2020, and public life slowly returned [25]. Further restrictions on public life were imposed on November 2, 2020, due to the persistence of the pandemic situation [5], which were then extended to a further lockdown on December 16, 2020 [6]. The first vaccines were made available to the German population on December 27, 2020, and the lockdown restrictions were eased again in Bavaria on March 8, 2021, depending on the incidence of new infections [26].

Reports from other medical fields showed a decrease in the number of patients presenting to the clinic during the lockdown periods and have discussed patients’ fear of COVID-19 infection during hospitalization as a possible reason for their reluctance [2, 22]. However, the number of patients presenting with abscesses remained the same during the first year of the COVID-19 pandemic in Germany. Furthermore, patients more often required an extraoral incision under general anesthesia (GA), however, without a prolonged inpatient stay.

Therefore, this study aimed to evaluate the first “COVID-19 year” in Germany, starting with the first lockdown phase (March 2020), as no restrictions were applied before, and compared the data of the first “COVID-19 year” with those of the two previous years (March 2018–March 2020).

## Methods

All clinical trials were conducted in accordance with the tenets of the Declaration of Helsinki. This retrospective study was approved by the Institutional Ethics Committee of the Technical University of Munich, Klinikum rechts der Isar (approval number: 227/21 S).

### Subjects

All consecutive cases with a diagnosis of odontogenic or intraoral abscess treated in an outpatient or inpatient setting were included in this study. Patients were divided into two groups: the control group, which included patients who first presented to our clinic between March 22, 2018, and March 21, 2020 (the two previous years), and the test group, which included all patients presenting between March 22, 2020, and April 10, 2021 (the first “COVID-19 year”). The date of March 22, 2021, was specifically chosen because it was the date when the first lockdown in Germany began, which lasted until May 3, 2020. The date of April 10, 2021, was the last day covered by the ethical approval mentioned. To assess the effect of age as a possible confounding factor, different groups were created (0–3 years for baby infants, 4–12 years for children, 13–64 years for adolescents and adults, 65–84 years for youngest-old and middle-old, and  ≥ 85 years for oldest-old). Since the age of  ≥ 65 years and a BMI  ≥ 40 kg/m^2^ (severe obesity) have been identified as risk factors for Covid-19 morbidity, these values were chosen as cut-offs to assess them as possible risk factors [13].

### Data collection

Data, including sex, age, type of abscess, treatment modality (GA vs. local anesthesia (LA); outpatient vs. inpatient), comorbidities, prior surgical or radio-/ chemotherapy treatment, medications, length of hospital stay (days), and healthcare costs (euro), were collected.

### Statistical analysis

All statistical analyses were performed using IBM SPSS Statistics, version 28 (SPSS Inc., Chicago, IL, USA). Graphs were generated using Excel (Microsoft Office version) and SPSS (version 28). Relative and absolute rates were calculated as descriptive statistics for nominal and ordinal variables. Mean and standard deviation, minimum and maximum, and median and interquartile range were calculated for metric parameters. The Shapiro–Wilk test and the Kolmogorov-Smirnoff test were used to test for normal distribution. Differences between groups were assessed using *t*-tests for normally distributed values or Mann–Whitney-U tests. Dependencies between categorical variables were analyzed using Chi-square or Fisher’s exact tests.

## Results

### Subjects

A total of 961 patients were included in this study. Of the 961 patients, 445 (46.3%) were females, and 516 (53.7%) were males. In the control group (*n* = 663), the gender distribution was quite similar (*n* = 146 females vs. *n* = 183 males in the year 2018–2019 and *n* = 147 females vs. *n* = 187 males in the year 2019–2020). In the test group (*n* = 298), the gender distribution converged with *n* = 152 females and *n* = 146 males (*p* = 0.051), and the number of patients was slightly smaller. Median age was in both groups not significant with 45 years in the control group and 42 years in the test group (range: 3–98 in both groups, *p* = 0.307, Mann–Whitney-U test), and the median length of hospital stay was 5 days (range: 1–51 days in the control group; range 1–50 days in the test group; *p* = 0.079, Mann–Whitney-U test). The recorded comorbidities are shown in Table 1. In the COVID-19 year, the number of patients with vascular diseases was significantly higher (*p* = 0.042), and the number of patients with epilepsy was significantly lower (*p* = 0.032) than those in the two previous years. The recorded possible risk factors are shown in Table 2. In the COVID-19 year, the rate of patients with a history of previous surgery was significantly higher (*p* = 0.015) than that in the two previous years. The rate of smoking was comparable between the test and control groups. The mean number of pack-years (py) was 19.8 (0.2–76.5 py; *p* = 0.177, Mann–Whitney-U test).

**Table 1:** Systemic diseases of the investigated cohort

**Systemic disease**	Preceding years *n* = 663 (%)	Covid-19 year *n* = 298 (%)	*p*-value
Allergy	164 (24.7)	83 (27.9)	
Lung disease total	65 (9.8)	32 (10.7)	
Asthma	40 (6.0)	19 (6.4)	
COPD	16 (2.4)	7 (2.3)	
Chronic bronchitis	3 (0.5)	3 (1.0)	
Tuberculosis	1 (0.15)	0 (0)	
Pneumonia	1 (0.15)	0 (0)	
COVID-19	0 (0)	2 (0.7)	
Others	4 (0.6)	1 (0.3)	
Non-medication-related bleeding disorders total	7 (1.1)	5 (1.7)	
Hemorrhagic diathesis Thrombophilia	4 (0.6)	2 (0.7)	
	3 (0.5)	3 (1.0)	
Diabetes mellitus total	52 (7.8)	31 (10.4)	
Diabetes mellitus type I	5 (0.8)	2 (0.7)	
Diabetes mellitus type II	47 (7.1)	29 (9.7)	
Cardiac disease total	86 (13.0)	46 (15.4)	
Coronary heart disease	54 (8.1)	16 (5.4)	
Myocardial Infarction	15 (2.3)	5 (1.7)	
Arrhythmia	36 (5.4)	24 (8.1)	
Pacemaker	9 (1.4)	3 (1.9)	
Cardiac insufficiency	24 (3.6)	15 (5.0)	
Conatal heart disease	0 (0)	2 (0.7)	
Valve replacement	6 (0.9)	5 (1.7)	
Hypertension	179 (27.0)	73 (24.5)	
**Vascular Disease**	42 (6.3)	30 (10.1)	0.042*
Liver/ bile disease	32 (4.8)	14 (4.7)	
Gastro-intestinal disease	60 (9.0)	25 (8.4)	
Kidney disease total	26 (3.9)	12 (4.0)	
Dialysis	4 (0.6)	1 (0.3)	
Thyroid disease	98 (14.8)	33 (11.1)	
**Epilepsy**	19 (2.9)	2 (0.7)	0.032*
Glaucoma	7 (1.1)	4 (1.3)	
Rheumatic disease	13 (2.0)	9 (3.0)	
Psychiatric disease	87 (13.1)	33 (11.1)	
Neoplasia total	64 (1.0)	31 (10.4)	
Benign	4 (0.6)	2 (0.7)	
Semimalignant	2 (0.3)	2 (0.7)	
Malignant after performed therapy	53 (8.0)	25 (8.4)	
Malignant	5 (0.8)	2 (0.7)	
Solid malignant head and neck tumor	16 (2.4)	6 (2.0)	
Solid malignant tumor elsewhere	33 (5.0)	21 (7.0)	
Haemato-oncological disease	9 (1.4)	5 (1.7)	

Preceding years = control group: 2018–2020

Covid-19 year = test group: 2020–2021

^*^Fisher’s Exact Test

**Table 2:** Distribution of possible risk factors of the investigated cohort

**Risk factors**	Preceding years *n* = 663 (%)	Covid-19 year *n* = 298 (%)	*p*-value
Wound healing disorders	18 (2.7)	6 (2.0)	
Smoker	197 (29.7)	83 (27.9)	
Alcohol (C2) consumption daily	18 (2.7)	10 (3.4)	
C2 occasionally	33 (5.0)	9 (3.0)	
C2 seldomly	1 (0.2)	0 (0)	
Drug history	16 (2.4)	2 (0.7)	
Pregnancy	4 (0.6)	2 (0.7)	
Age 0–3 years	1 (0.2)	1 (0.3)	0.136**
Age 4–12 years	25 (3.8)	11 (3.7)	
Age 13–64 years	495 (74.7)	225 (75.5)	
Age 65–84 years	127 (19.2)	46 (15.4)	
≥ 85 years of age	15 (2.3)	15 (5.1)	
≥ 65 years of age	142 (21.4)	61 (20.5)	
Long-term care^a^			
Long-term care nursing home	20 (3.3)	9 (3.2)	
Long-term care outpatient service	4 (0.7)	0 (0)	
BMI ≥ 40 kg/m^2b^	4 (1.5)	5 (3.2)	
Previous surgery^c^	274 (55.0)	136 (65.1)	*0.015
≥ 5 drugs for medication	92 (13.9)	41 (13.8)	
Antiresorptive drugs total	18 (2.8)	9 (3.0)	
Current intake	17 (2.6)	8 (2.7)	
History of intake	1 (0.2)	1 (0.3)	
Glucocorticoids	18 (2.7)	12 (4.0)	
Immune suppressive therapy	34 (5.1)	17 (5.7)	
ACE-I, ARB	93 (14.0)	46 (15.4)	
NSAID	147 (22.2)	61 (20.5)	
History of radiation therapy	14 (2.1)	5 (1.7)	

Preceding years = control group: 2018–2020

Covid-19 year = test group: 2020–2021

*Abbreviations*: *ACE-I* Angiotensin-converting enzyme (ACE) inhibitors, *ARB* Angiotensin receptor blockers, *NSAID* Non-steroidal anti-inflammatory drugs

^a^*n* = 876 (85 non-registered cases)

^b^*n* = 707 (254 non-registered cases)

^c^*n* = 461 (545 non-registered cases)

^*^Fisher’s Exact Test

^**^Chi^2^-Test

### Clinical findings

In the COVID-19 year, only two out of 123 PCR-tested patients tested positive for COVID-19 and were treated as inpatients with special isolation precautions. The rate of patients with poor oral hygiene and a desolate dental chart was significantly higher in the COVID-19 year than that in the previous two years (*p* < 0.001). A statistically significant difference in the progression of abscesses was observed between the test and control groups. In the control group (two previous years), there were more paramandibular abscesses, whereas in the test group (the COVID-19 year) there were relatively and statistically significantly more perimandibular abscesses (*p* = 0.002*). The clinical findings are summarized in Table 3 and potentially life-threatening situations (in total 25 out of 26 ICU patients) are also listed in Table 3. In addition to odontogenic abscesses, intraoral abscesses were also found to be the result of cancerous lesions, osteoradionecrosis, sialolithiasis, postoperative / post biopsy wound infections, drug-induced related osteonecrosis of the jaw, osteomyelitis, infected augmentation material, and traumatic bites.

**Table 3:** Clinical findings comparing Covid-19 year with the two preceding years regarding abscess localization, presence of desolate dental status, length of hospitalization and ICU stay, and time to treatment

**Clinical findings**	**Preceding years** ***n* = 663 (%)**	**Covid-19 year** ***n* = 298 (%)**
**Abscess type and location**		
Pericoronitis-abscess	5 (0.8)	1 (0.3)
Apical periodontitis/ Periimplantitis-abscess	23 (3.5)	14 (4.7)
Enosseal abscess	1 (0.1)	0 (0)
Subperiosteal abscess	18 (2.7)	17 (5.7)
Submucosal abscess	52 (7.8)	16 (5.4)
Fossa canaina abscess	130 (19.6)	64 (21.5)
Parotid abscess	1 (0.1)	0 (0)
Lip abscess	4 (0.6)	0 (0)
Tongue abscess	2 (0.3)	0 (0)
Palatinal abscess	5 (0.8)	5 (1.7)
Buccal abscess	45 (6.8)	19 (6.4)
Retromaxillary abscess	0 (0)	2 (0.7)
Chin abscess	5 (0.8)	0 (0)
Paramandibular abscess	170 (25.6)	57 (19.1)
Perimandibular abscess	91 (13.7)	61 (20.5)
Sublingual abscess	26 (3.9)	9 (3.0)
Submental abscess	13 (2.0)	6 (2.0)
Submandibular abscess	35 (5.3)	8 (2.7)
Masseterico-/pterygomandibular abscess	15 (2.3)	6 (2.0)
Para-/retropharyngeal abscess	3 (0.5)	1 (0.3)
Several lodges abscess	1 (0.1)	3 (1.0)
Not other specified abscess	18 (2.7)	9 (3.0)
**Desolate dental chart**	171 (25.8)	109 (36.6)
**Hospitalisation (days)**	mean 6 (1–51)	mean 6 (1–50)
**Length of ICU stay (days)**	mean 10 (1–27)	mean 5 (1–12)
**Time to treatment (days)**	mean 7 (1–270)	mean 8 (1–320)
**Potentially life-threatening cases** **(ICU patients) *n* = 25**	**Preceding years** ***n* = 17 (68)**	**Covid-19 year** ***n* = 8 (32)**
Massive pharyngeal swelling (potentially life-threatening)	11 (44)	7 (28)
Dysphagia	1 (4)	0 (0)
Oxygen saturation drop	2 (8)	0 (0)
Acute kidney disease	0 (0)	1 (4)
Mediastinitis	1 (4)	0 (0)
Sepsis	2 (8)	0 (0)

### Treatment modality

**Fig. 1:**
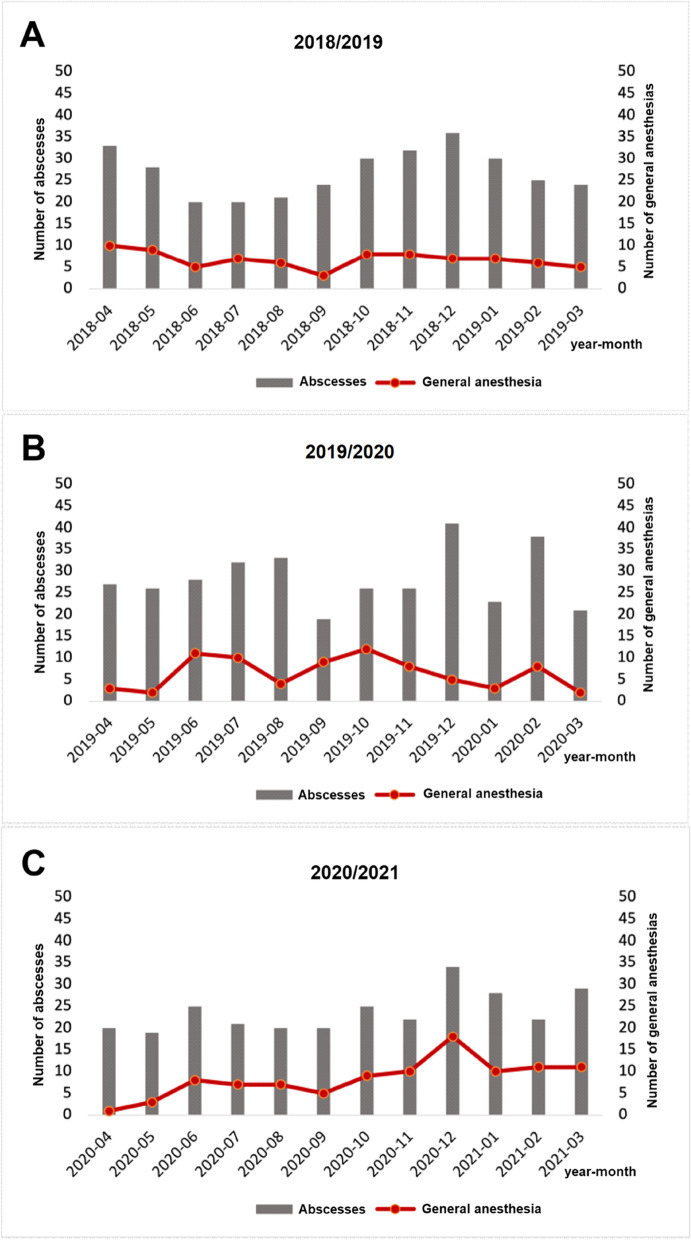
Rate of abscess incisions. Grey: total number; red: GA. **A** year 2018/19; **B** year 2019/20; **C** year 2020/21

**Fig. 2:**
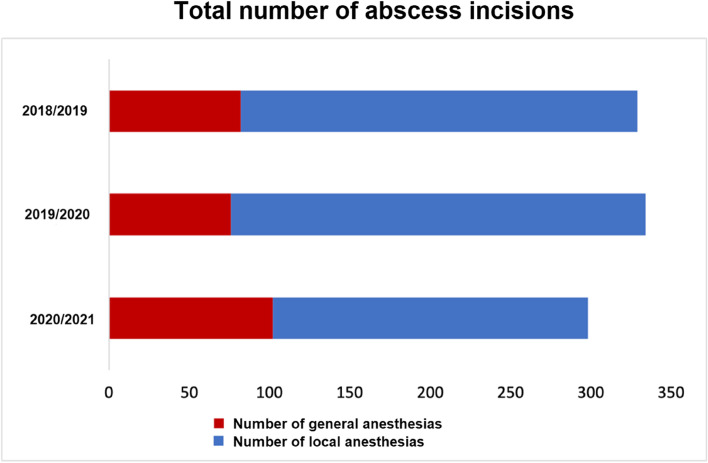
Distribution of LA and GA over the three years. Red: GA; blue: LA

**Fig. 3:**
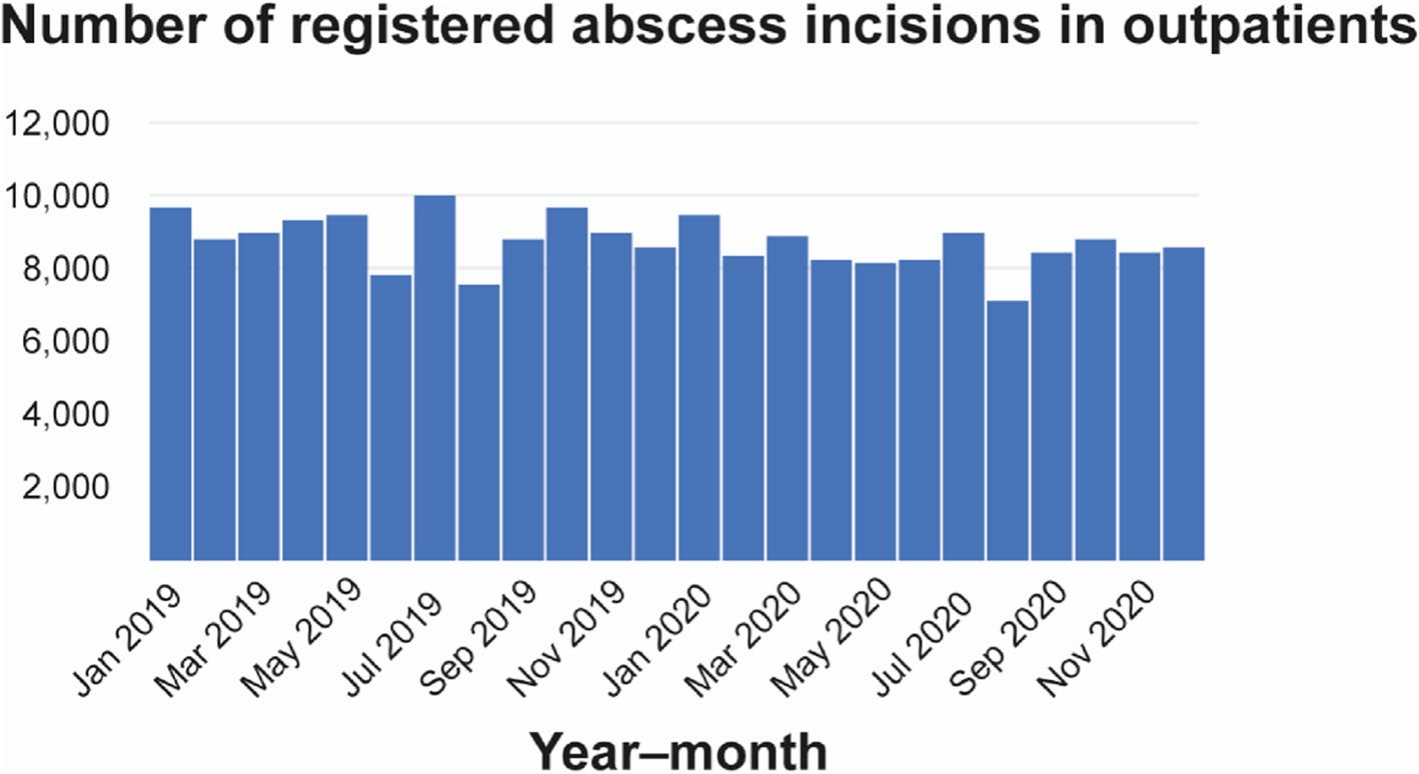
Number of abscess incisions in outpatient setting with monthly breakdown. Data based on the billing code Ä116, kindly provided by the Kassenzahnärztliche Vereinigung (20.04.2021)

Regarding invasiveness, four groups were formed: GA including mask anesthesia with inpatient stay, GA with outpatient stay, LA with inpatient stay, and LA with inpatient and outpatient stay. Neither in the COVID-19 year nor in previous years were outpatients treated under GA, including mask anesthesia. A statistically significant difference was observed between the test group and the control group regarding the invasiveness of the operation/anesthesia. Therefore, differences between the two groups were calculated for GA and LA with statistically significant findings (*p* < 0.001; Table 3), with numbers increasing at the end of the first block (Fig. 1). Figure 2 shows the different distribution of cases in COVID-19 year and the two previous years, and Fig. 3 shows the number of odontogenic and intraoral abscesses in the state of Bavaria per month for the whole period. The rate of GA cases was similar in the two previous years (2018–2019 (24.9%) and 2019–2020 (22.8%)) and showed a remarkable increase in the COVID-19 year (34.2%). As a result, statistically significantly more patients in the test group were treated as inpatients than in the control group (*p* < 0.001). The rate of patients treated with LA who required postinterventional hospitalization increased slightly (17.6% vs. 19.8%, Table 4).

**Table 4:** Comparison of Covid-19 year with the two preceding years regarding therapy modality (general anesthesia/local anesthesia) and in-/outpatient treatments

n (%) total (961)	Therapy modality	Outpatients	Inpatients	Total	*p*-value
Preceding years	LA	388	117	505 (52.6)	< 0.001*
	GA	0	158	158 (16.4)	
	Total	388 (40.0)	275 (28.6)	663 (68.7)	
Covid-19 year	LA	137	59	196 (20.4)	
	GA	0	102	102 (10.6)	
	Total	137 (14.3)	161 (16.8)	298 (31.0)	
Total		525 (54.6)	436 (45.4)	961 (100)	

Preceding years = control group: 2018–2020

Covid-19 year = test group: 2020–2021

*Abbreviations*: *LA* local anesthesia, *GA* general anesthesia

^*^Fisher’s Exact Test

No significant difference in ICU stay was observed between the control (2.7%) and test groups (3.0%) (*p* = 0.834). The average length of stay in the ICU also showed no significant difference between the control (10 days) and test groups (5 days) when one outlier was removed (*p* = 0.200, Mann–Whitney-U test).

### Time to treatment

The time to treatment for 793 patients was recorded: 556 in the control group and 237 in the test group. The mean time to treatment was 6.67 (± 17.3) days in the control group and 8.30 (± 27.5) days in the test group, without statistical significance between the groups (*p* = 0.194, Mann–Whitney-U test).

### Economical aspects

The increase in diagnosis-related group-based revenue was calculated in the COVID-19 year compared to the average of the two previous years. The average cost of outpatient treatment in the two previous years was €646,481 per year, whereas it increased to €746,895 in the COVID-19 year. For outpatients, the increase in the COVID-19 year compared to the control group was + 15.5%, while for inpatients, it was + 21.3%, from an average cost of €557,685 per year in the two previous years to €676,549 in the COVID-19 year. Follow-up care after discharge was included in the outpatient costs. All costs were absolute costs and included increases in inpatient costs between 1.9% and 3.6% per year. However, no significant difference in the length of inpatient stay was observed (*p* = 0.79). The mean length of stay was 6 (± 5) days in the control group and 6 (± 6) days in the test group.

## Discussion

The outbreak of COVID-19 required many adjustments for the population and the healthcare system. For the first time in recent history, the response to a pandemic crisis in the Federal Republic of Germany led to a lockdown. These novel pandemic situations had an impact on disease management, as evidenced by delays in cancer diagnosis [17, 24] and as suggested by our example of abscess formation. In addition, the pandemic situation had a serious impact on civilian life, as evidenced by data on increasing numbers of physical assault-related traumas and in specific domestic violence-related traumas during the Italian lockdown show [8, 10]. The authors interpret their findings as a result of social isolation during the pandemic, especially during the lockdowns.

The data from our study showed that patients presenting to our clinic had a higher percentage of advanced stages of their odontogenic abscesses compared to the previous two years, which can be deduced from the increased rate of abscess incisions under GA. These results reached statistical significance. In our department, the indication for GA is always assessed by an experienced senior consultant, guaranteeing the same quality standard throughout the observation period.

A similar trend was observed by Parara et al. in Athens, who reported a significant increase in descending necrotizing mediastinitis. The focus of the healthcare system on COVID-19 was also mentioned as a possible reason, with patients staying away from hospitals for fear of COVID-19 [19]. Yakubov et al. argued that the limited dental service available and the focus on emergencies only in the early period of COVID-19 might have discouraged patients from coming to the clinic, resulting in more severe cases requiring treatment [32]. Dawoud et al. also studied the first period of the lockdown from March to June 2020. Their data showed an unfavorable situation, with fewer patients with odontogenic infections being treated during this period [7]. The same trend was observed by Dang et al. and Johnson et al. In their departments in France and England, respectively, the closure period covered approximately the same period (March to May/June 2020). Compared to the previous years, they observed fewer serious dental infections [13, 20]. However, possibly reluctant patients may not have been considered when observing a rather short period due to a possible delay in the onset of chronic apical infection to the progression of abscess formation, as seen in our patients requiring GA in June, July, and December 2020 (Fig. 1C).

Another study conducted in France, with a longer observation period from March to December 2020, reported a general decrease in the number of patients presenting with facial inflammation during the first year of the pandemic [15]. Although we also see a slight decrease in the number of cases (compared to the two previous years: mean 10%), the two studies are not fully comparable, as our study focuses more on abscess formation and treatment modality. While Seppänen et al. observed a shift toward more invasive dental infections in the facial and cervical regions within a decade in the past (1994 vs. 2004), the rapid increase in the number of GA required in the test group within one year cannot be explained by this possible general trend and is more likely a consequence of the pandemic situation [23]. When we asked our patients about the reasons for their late presentation, many patients mentioned fear of COVID-19 infection during their inpatient stay or strict regulations when entering a hospital. This possibility of fear of infection was also discussed as a reason for late presentation in a study by Kün-Darbois et al. [15].

Interestingly, the increase in the need for GA in the COVID-19 cohort showed a similar trend to the increase in COVID-19 infection cases in Germany, with a delay of about one month [21]. According to the billing standard in the state of Bavaria, which we officially requested, the registered billing figures for intraoral abscess incisions in an outpatient setting remained at a relatively similar level at the beginning of the pandemic and during the first year [14]. However, the increased rate of GA and subsequent inpatient treatment, using the example of advanced oral abscesses, resulted in further costs and financial burdens on the healthcare system, including cancellations of planned surgery and compensation payments to hospitals [29].

It is explicitly not the intention of the authors to evaluate or question the strategies and measures taken to control the COVID-19 pandemic. However, it is possible that future global pandemics may occur [16]. In addition, the measure of lockdowns may have led to social isolation with an impact on the incidence of domestic trauma, as to be observed in the Italian studies mentioned above, but it also to a possible delay in the treatment of dental diseases, leading to advanced abscess formation, as suggested by this study. Sequestration as a pandemic control measure may also have delayed the treatment of odontogenic and intraoral abscess formation, as shown by the main results of our study. Therefore, one lesson that could be drawn from the results of this and other studies is that people with medical concerns need to be encouraged to seek help in a timely manner. On the one hand, these basic health services need to be guaranteed and accessible without any restrictions. This requires widespread education of the population, using social media as well as traditional media such as newspapers, radio and television, about the necessary check-ups and the urgent medical conditions that need to be treated at all times. On the other hand, these basic medical services must be unconditionally guaranteed and accessible. In addition to the above-mentioned studies, which were published very early in 2020 but covered only a short period of the COVID-19 period, this study evaluated the first year, including the pandemic lockdown of the healthcare system and civil life. Therefore, our study provided an additional overview and showed possible conflicts in our healthcare system during the pandemic, especially during lockdowns, which should be considered in possible future healthcare crises.

This study has some limitations. First, the retrospective design of this study is a limitation. Second, the subsequent years of the pandemic were not included in this study. However, this was intended as the restrictions were gradually relaxed, and regional differences made an effective analysis impossible. Third, the costs evaluated for outpatient care were not aggregated due to a bundled payment for patients treated at universities, so, unlike inpatient care, costs had to be calculated by rounding for all groups.

## Conclusion

During the first year of the COVID-19 pandemic in Germany, the rate of severe odontogenic infections with abscess formation increased significantly at our institution. This disease progression significantly increased the need for general analgesia, with a corresponding increase in costs to the healthcare system. The focus on COVID-19 may have caused additional healthcare problems, as illustrated by the example of intraoral abscesses.

## Data Availability

Data used for analysis is made available and is presented in the tables.
